# Supplementary material for: Molecular interplay of ASNS and the PI3K-AKT-mTOR pathway in CMV and HIV co-infections: Therapeutic implications
Source: PLoS One. 2026 Feb 20;21(2):e0342050. doi: 10.1371/journal.pone.0342050 (PMC12923000; doi:10.1371/journal.pone.0342050)
Supplement: Attachment — Submitted filename: Peer Review Report-Molecular.docx [file pone.0342050.s004.docx]

Peer Review Report

**Title: Molecular interplay of ASNS and the PI3K-AKT-mTOR pathway in CMV and HIV co-infections: therapeutic implications**

**Peer Reviewer**: Luqman Abass

**1. Originality and Novelty**

The manuscript presents the results of original research on the interplay between ASNS and the PI3K-AKT-mTOR pathway in CMV and HIV co-infections. This is a novel approach, as few studies have explicitly examined ASNS in this context. The work employs an integrative bioinformatics approach to analyze gene expression datasets, single-cell RNA sequencing, and molecular docking studies.

**2. Prior Publication**

The study results, as presented, appear to be unpublished elsewhere. The manuscript does not reference any previous work by the authors that might suggest duplicate publication. Additionally, the findings integrate various datasets in a unique way.

**3. Technical Rigor: Experiments, Statistics, and Analyses**

Bioinformatics Analysis: The study applies multiple bioinformatics techniques, including differential expression analysis, protein-protein interaction (PPI) network analysis, and enrichment analysis. The use of publicly available datasets is appropriate, though the absence of direct co-infected patient data is noted as a limitation.

Single-Cell RNA Sequencing: The methodology for filtering and normalizing scRNA-seq data is sound. The annotation of plasma cells using marker genes (IGJ, IGLL5, MZB1) aligns with standard practices.

Machine Learning: The use of the Random Forest algorithm to assess the importance of ASNS and transcription factors is well justified. The authors provide a robust explanation of their feature selection criteria.

Molecular Docking and Dynamics: The molecular docking study is well conducted using AutoDock Vina, and molecular dynamics simulations extend the analysis by assessing ligand stability.

Statistical Analysis: The authors provide appropriate statistical tests, including adjusted p-values for multiple comparisons. However, some methodological details (e.g., the exact number of independent runs in docking) could be further clarified.

**4. Data and Conclusions: Appropriateness and Support**

The manuscript presents conclusions logically derived from the data. ASNS is consistently identified as a hub gene across multiple analyses, supporting its proposed role.

The molecular docking studies suggest a potential therapeutic role for cidofovir, though experimental validation is necessary.

The transcription factor analysis highlights RUNX1, ATF4, and MDM2 as key regulators, providing a plausible mechanistic framework.

While the conclusions are well-founded, the discussion does not fully address potential alternative explanations or limitations in dataset selection.

**5. Clarity, Language, and Readability**

The manuscript is written in clear and standard English, with a logical structure. Figures and tables are well integrated, aiding in comprehension.

**6. Ethical Standards and Research Integrity**

The manuscript states that it relies solely on published datasets, eliminating concerns about direct ethical approvals.

There are no conflicts of interest declared.

The study complies with standard bioinformatics data-sharing and reporting guidelines.

**7. Adherence to Community Standards**

The manuscript follows appropriate reporting guidelines for bioinformatics research.

The inclusion of supplementary materials (e.g., DEGs lists) ensures data transparency.

The methodology is well documented, allowing reproducibility.
