# Supplementary material for: Molecular interplay of ASNS and the PI3K-AKT-mTOR pathway in CMV and HIV co-infections: Therapeutic implications
Source: PLoS One. 2026 Feb 20;21(2):e0342050. doi: 10.1371/journal.pone.0342050 (PMC12923000; doi:10.1371/journal.pone.0342050)
Supplement: Attachment — Submitted filename: Response to Reviewers.docx [file pone.0342050.s005.docx]

Dear Editors and Reviewers,

We sincerely appreciate your constructive feedback and insightful suggestions, which have significantly enhanced the quality of our manuscript. Your time and effort in reviewing our work are invaluable, and we are deeply grateful for the opportunity to improve our research presentation.

**### Response to Reviewer #1**

Thank you for your positive evaluation and recognition of our study's potential contributions to CMV and HIV therapeutic strategies.

**Comment:** Reviewer #1 provided a general positive assessment of the study.

**Response:** We are sincerely grateful for your positive feedback and assurance that our study aligns with high scientific standards. Your recognition of the robust analyses and potential implications of our findings is highly encouraging. We are confident that our work will contribute valuable insights to the field.

**### Response to Reviewer #2**

Thank you for your detailed review and valuable suggestions to strengthen our study.

**Question 1:** Reviewer #2 suggested adding a comparison with known ASNS inhibitors and including literature references to strengthen the discussion.

**Response 1:** We fully agree with your suggestion. To address this, we have incorporated a comparison of the binding efficiency of ONL and Cidofovir with known ASNS inhibitors such as Bisabosqual A into the Results section (Fig. 7A-D). Relevant literature references have also been added to provide a broader context for our findings (Discussion section, line 554).

**Question 2:** Reviewer #2 recommended incorporating molecular mechanics-based free energy calculations (MM/PBSA or MM/GBSA) to further validate the stability and binding affinity of the ligands.

**Response 2:** We acknowledge the importance of MM/PBSA or MM/GBSA calculations for validating the binding affinity and fully agree with your suggestion. We have used two docking programs as complementary approaches to enhance the reliability of our results. However, due to the significant computational resources and time required for these calculations, which our current laboratory setup cannot accommodate, we were unable to include them in this revision. We have discussed this limitation in the discussion section and emphasized that incorporating these calculations will be a priority for our future work (see discussion section, line 560). We sincerely appreciate your understanding and valuable suggestion, and we will definitely continue to submit our future work to PLOS ONE.

**Question 3:** Reviewer #2 suggested discussing the functional role of key residues (VAL-52, VAL-51, ARG-48) in ASNS activity and their potential impact on enzyme function or substrate binding.

**Response 3:** We appreciate this suggestion and have expanded our discussion to include the functional roles of these key residues in ASNS activity. We have detailed how these residues are involved in substrate binding and catalysis, and how interactions with our ligands may influence these processes (Discussion section, line 556).

**### Response to Reviewer #3**

Thank you for your thorough review and specific suggestions to improve our manuscript.

**Question 1:** Reviewer #3 requested an explanation of how ASNS modulates the PI3K/AKT/mTOR pathway.

**Response 1:** We have conducted a co-expression analysis and used machine learning to screen for key interacting molecules. These analyses have been added to the discussion section to provide a detailed explanation of the molecular mechanisms by which ASNS interacts with the PI3K/AKT/mTOR pathway. Specifically, we have illustrated how ASNS may modulate the pathway through its interactions with key molecules such as AKT2 (lines 564-570).

**### Additional Modifications and Clarifications**

In addition to addressing the reviewers' comments, we have made several clarifications and modifications to enhance the quality of our manuscript:

1. **Renaming Microglia to Pro-inflammatory Macrophages**: In our single-cell analysis, we renamed Microglia to pro-inflammatory macrophages to better reflect their role in chronic inflammation and viral infections (Results section, line 390).

2. **Role of the Funding Agency**: We have added a statement in the acknowledgments section to clarify the role of the funding agency (Funding Statement section).

3. **Code Availability**: We have added a statement in the data availability section indicating that the code used in our study can be obtained from the corresponding author upon request (Data Availability section).

4. **Docking Procedures**: We have clarified these details in the Methods section, specifying the number of independent runs performed during the docking experiments to ensure reliability and reproducibility (line 218).

5. **Limitations of Dataset Selection**: We have expanded the Discussion section to address the limitations of the datasets used in our study and provide a more comprehensive interpretation of our results (line 571).
6. **Update on Author Affiliation**：Due to a change in the workplace of the first author,Hao Zhang, his affiliation has been updated to:**Geriatrics Center, Wuxi Second Geriatric Hospital, Wuxi, Jiangsu Province, China**.
7. **Regarding Figure Submission and PACE Access Issue**:

We attempted to access the PACE website to check our figures as suggested but were unable to open the link provided. However, we have already revised and optimized our figures, ensuring a consistent style and high-quality presentation. Should there be any further requirements or modifications needed, we are readily available to make the necessary adjustments. Thank you for your understanding and support.

**### Submission Details**

We have submitted two versions of our manuscript: a complete revised version and a version with modifications highlighted in red for your convenience.

Once again, we express our deepest gratitude to the reviewers for your invaluable feedback and constructive suggestions. We are truly thankful for the time and effort you've dedicated to reviewing our work, and we sincerely appreciate the editors' guidance and support throughout this process.

Thank you for your time and consideration.

Best regards,

Wei Lu
