# Supplementary material for: Molecular interplay of ASNS and the PI3K-AKT-mTOR pathway in CMV and HIV co-infections: Therapeutic implications
Source: PLoS One. 2026 Feb 20;21(2):e0342050. doi: 10.1371/journal.pone.0342050 (PMC12923000; doi:10.1371/journal.pone.0342050)
Supplement: Attachment — Submitted filename: Response_to_Reviewers_auresp_2.docx [file pone.0342050.s006.docx]

**Response to Reviewers**

Dear Reviewers,

We deeply appreciate your valuable time, professional insights, and constructive feedback, which have significantly enhanced our manuscript’s scientific rigor, clarity, and depth. Below are detailed responses to each comment, with revisions implemented in the second draft.

**Response to Reviewer 2**

Thank you for your positive assessment of our revised manuscript and recognition of our efforts to address first-round review issues, including analyses of ASNS inhibitors and key binding sites. We explicitly acknowledge the limitation of not performing MM/PBSA or MM/GBSA calculations in the Discussion section to ensure scientific transparency. We are grateful for your support and publication recommendation.

**Response to Reviewer 4**

Your comprehensive insights have been pivotal to refining our manuscript. We have addressed each concern in detail below:

**2. Abstract**

**2.I Question: The biological characteristics, clinical significance, and disease exacerbation mechanisms of CMV/HIV coinfection were not explained. To highlight the research urgency, it is recommended to add a sentence illustrating the clinical significance of CMV/HIV coinfection and the deficiencies in current mechanistic research.**

Answer: Thank you for this critical suggestion. The revised abstract opens with a sentence clarifying the clinical burden of CMV/HIV coinfection and unmet mechanistic research needs, directly addressing this gap.

Location in Revised Manuscript: Abstract, Lines 17-19

**2.II Question: Need to clearly define the association between ASNS as a metabolic/signaling hub and the PI3K-AKT-mTOR pathway to highlight innovation.**

Answer: We appreciate your insight. The revised abstract positions ASNS as a "pivotal metabolic-signaling hub" with "critical interactions with the PI3K-AKT-mTOR pathway," while highlighting machine learning validation of AKT2 as a key node to emphasize innovation.

Location in Revised Manuscript: Abstract, Lines 19-21

**2.III Question: The therapeutic significance section is vague. Although the binding of cidofovir to ASNS is valuable, its clinical relevance or theoretical hypothesis is not clarified. Need to strengthen the logical basis and adopt a hypothesis-driven approach.**

Answer: Thank you for pointing out this ambiguity. We revised this section to frame the finding as hypothesis-driven, specifying cidofovir as an "approved antiviral agent" and linking its high-affinity binding to ASNS to a potential host-directed therapeutic strategy, without overstating clinical applicability.

Location in Revised Manuscript: Abstract, Lines 31-35

**2.IV Question: It is recommended to conclude from a translational medicine perspective, e.g., "In summary, our findings indicate that ASNS is a metabolic-signaling hub exploited during viral coinfection, highlighting its potential as a novel therapeutic target. This study lays the groundwork for subsequent experimental validation and the development of host-directed therapeutic strategies against CMV-HIV coinfection."**

Answer: We are grateful for your guidance. The revised abstract’s conclusion aligns closely with your suggestion, emphasizing ASNS’s therapeutic potential and laying the groundwork for future validation and host-directed therapy development.

Location in Revised Manuscript: Abstract, Lines 35-38

**3. Introduction**

**3.I Question: This section is overly descriptive with insufficient mechanistic discussion, resembling a review rather than an original research introduction. It is recommended to propose an innovative biological hypothesis early, starting with the importance of coinfection. For example, not only describe the prevalence but also emphasize the synergistic pathogenic mechanisms (e.g., CMV reactivation under HIV infection exacerbates immunosuppression, leading to increased non-AIDS-related morbidity).**

Answer: Thank you for this constructive critique. The revised introduction prioritizes mechanistic discussion, highlights the synergistic pathogenic cycle of HIV and CMV, and explicitly states our hypothesis—that ASNS acts as a central metabolic-signaling hub—early in the section.

Location in Revised Manuscript: Introduction, Lines 40-44; Lines 65-66

**3.II Question: It is recommended to supplement regional difference data on the 90% coinfection prevalence or compare the prevalence before and after antiretroviral therapy (ART).**

Answer: We appreciate your suggestion. We retained the well-cited 90% seroprevalence and contextualized it within HIV-induced immunosuppression (a cross-regional, ART-relevant mechanism), ensuring it highlights clinical significance without diverting from core molecular research.

Location in Revised Manuscript: Introduction, Line 40

**3.III Question: Need to more clearly identify the research gap. For example, explicitly state that although HIV and CMV each hijack host metabolic and signaling pathways, the shared metabolic reprogramming mechanisms during coinfection remain unclear.**

Answer: Thank you for urging clarity on the research gap. The revised introduction explicitly states that shared molecular mechanisms driving HIV/CMV synergistic pathogenesis remain undefined, directly addressing this point.

Location in Revised Manuscript: Introduction, Lines 50-52

**3.IV Question: The introduction of ASNS as a "key gene" is abrupt without sufficient prior biological context. It is recommended to first summarize ASNS’s roles in metabolic regulation, viral pathogenesis, and immune modulation, then justify why it may be a critical vulnerable node in coinfection.**

Answer: We are grateful for identifying this abrupt transition. The revised introduction provides context on ASNS’s roles in amino acid metabolism, viral replication, and PI3K-AKT-mTOR interaction, ensuring a logical link to its proposed role in coinfection.

Location in Revised Manuscript: Introduction, Lines 56-59

**3.V Question: It is recommended to rephrase the rationale for bioinformatics analysis. For example, replace "cost-effective" with emphasizing that integrating transcriptomics and network analysis enables unbiased discovery of shared molecular nodes during infection.**

Answer: Thank you for this refinement. We revised the bioinformatics rationale to emphasize "unbiased identification of shared molecular nodes" (instead of cost-effectiveness), highlighting scientific strength.

Location in Revised Manuscript: Introduction, Lines 72-74

**[3.VI](3.VI) Question: Although the PI3K-AKT-mTOR pathway is mentioned, it is not clarified how CMV and HIV hijack this pathway. It is recommended to elaborate on the pathway in the context of viral infection, e.g., explaining that both viruses exploit this pathway for replication, survival, and immune evasion, thereby positioning ASNS as an upstream regulator of this pathway.**

Answer: We appreciate your guidance. The revised introduction clarifies that both viruses hijack the PI3K-AKT-mTOR pathway for survival and replication, and positions ASNS as a potential mediator of this process.

Location in Revised Manuscript: Introduction, Lines 58-59

**4. Materials and Methods**

**4.I Question: Some datasets (e.g., GSE14490, GSE68563, GSE6740) are repeatedly introduced in different contexts, leading to redundancy and confusion. It is recommended to present dataset information uniformly in a table (e.g., dataset ID, tissue type, experimental conditions, sample size) and reference the table in subsequent sections.**

Answer: Thank you for identifying this redundancy. We added Table 1 to summarize all GEO datasets, with subsequent methods sections referencing the table to streamline the narrative.

Location in Revised Manuscript: Introduction, Lines 75-78 (Table 1); Materials and Methods sections (all dataset references link to Table 1)

**4.II Question: Although the computational method details are abundant, the biological rationale for each step is often unclear. For example: Why choose the random forest algorithm over other machine learning methods (for prioritizing network nodes)? Why focus on plasma cells (are they HIV infection reservoirs or effector cells)? Why select cidofovir as a ligand (does its known antiviral activity suggest off-target host effects)?**

Answer: We appreciate your question on methodological rationale. We added justifications: 1) Random forest for robustness in high-dimensional data; 2) Plasma cells for HIV immune relevance; 3) Cidofovir for clinical approval and predicted ASNS-binding activity.

Location in Revised Manuscript: Bioinformatics Analysis section, Lines120-124; Single-Cell RNA Sequencing Analysis section, Lines 163-167; Molecular Docking Analysis section, Lines 223-224

**4.III Question: Some datasets have small sample sizes (e.g., 3,000 single-cell RNA sequencing cells, limited samples for leave-one-out cross-validation). Need to acknowledge or emphasize this limitation in the methodology or discussion section.**

Answer: Thank you for reminding us to address sample size limitations. We acknowledged these in methods and discussion sections, noting mitigation via leave-one-out cross-validation.

Location in Revised Manuscript: Analysis of ASNS and the PI3K-AKT-mTOR Pathway in CMV Infection section, Lines 112-113; Single-Cell RNA Sequencing Analysis section, Line 152; Discussion section, Lines 592-601

**4.IV Question: There is a lack of integration between subsections. It is not clear how data from different levels collectively point to ASNS as a hub molecule. It is recommended to add an introductory paragraph in the methodology section to integrate the analysis workflow, e.g., "This study combines transcriptomic analysis, network analysis, machine learning, single-cell RNA sequencing, and molecular modeling to identify host factors associated with CMV and HIV coinfection. Analyses at different levels progressively converge to identify ASNS as a candidate therapeutic node."**

Answer: Thank you for this suggestion. We added an introductory paragraph to Materials and Methods, outlining how integrated analyses (transcriptomics, machine learning, etc.) converge on ASNS as a key hub.

Location in Revised Manuscript: Materials and Methods, introductory paragraph (before subsection 1), Lines 85-87

**5. Results**

**5.I Question: The description of figures is overly detailed, leading to lengthy and mechanical narration without conceptual refinement. The results section should focus on highlighting biological significance rather than describing technical details of each violin plot or RMSD change. For example, replace descriptions of "each violin plot or RMSD change" with: "Analysis of multiple CMV and HIV datasets shows consistent upregulation of ASNS and strong co-expression with PI3K-AKT-mTOR pathway components (especially AKT2), suggesting a shared metabolic-signaling axis activation during coinfection."**

Answer: Thank you for this feedback. The revised results focus on biological conclusions (e.g., ASNS upregulation, AKT2 co-expression) rather than detailed figure descriptions, emphasizing conceptual insights.

Location in Revised Manuscript: All Results subsections (e.g., Differential Gene Expression and Functional Enrichment Analysis section, Lines 265-275; Expression Analysis of ASNS and Key Molecules section, Lines 288-291)

**5.II Question: There is redundant expression in subsections. For example, the idea of "ASNS upregulation and co-expression with PI3K-AKT-mTOR" is repeated across different datasets. It is recommended to integrate redundant descriptions and present findings comprehensively across datasets. For example, avoid describing ASNS co-expression in CMV and HIV in three separate paragraphs and instead merge the analysis.**

Answer: We appreciate your observation. We integrated redundant findings into a single subsection, comprehensively presenting ASNS expression patterns across CMV and HIV datasets to eliminate repetition.

Location in Revised Manuscript: Differential Expression of ASNS and PI3K-AKT-mTOR Pathway Components in CMV and HIV Infections section, Lines 309-351

**5.III Question: All bioinformatics and in silico simulation results should explicitly use "correlation" rather than "causal relationship" expressions. For example, although molecular docking results suggest an interaction between cidofovir and ASNS, without functional validation, it is premature to imply the feasibility of drug repurposing, and these results should be positioned as hypothesis-generating.**

Answer: Thank you for emphasizing scientific rigor. We revised all in silico results to use correlative, hypothesis-generating language (e.g., "potential interaction," "preliminary framework") to avoid overinterpretation.

Location in Revised Manuscript: Binding Affinity and Interaction Analysis section, Line 491; Molecular Dynamics Analysis section, Line 521-526

**5.IV Question: The research on immune checkpoint-related findings is insufficient. For example, LAG3/ITPRIPL1 changes are mentioned but not mechanistically integrated with the ASNS/PI3K-mTOR pathway, and the biological mechanisms of the signaling pathway are underdiscussed. The results section repeatedly mentions correlations but does not explain how ASNS regulates the PI3K-AKT-mTOR pathway in the context of HIV/CMV coinfection. It is recommended to strengthen biological mechanism interpretation, e.g., Does ASNS-driven amino acid biosynthesis provide energy for the PI3K-AKT-mTOR signaling pathway? Does this represent a metabolic vulnerability in viral coinfection? Is LAG3 upregulation a compensatory immune checkpoint response to ASNS/PI3K-mTOR activity?**

Answer: We are grateful for your guidance. The revised results link LAG3 upregulation to ASNS-PI3K-AKT-mTOR activation, proposing ASNS-driven amino acid biosynthesis as a metabolic vulnerability, framed as testable hypotheses.

Location in Revised Manuscript: Expression Analysis of ASNS and Key Molecules section, Lines 301-312; Differential Expression of ASNS and PI3K-AKT-mTOR Pathway Components section, Lines 340-349

**6. Discussion**

**6.I Question: This section is overly descriptive and redundant, with some content resembling a restatement of results (e.g., volcano plots, docking hydrogen bonds, machine learning details) rather than conceptual integration. It is recommended to shift the focus from description to conceptualization, avoiding repeating docking results and instead emphasizing their biological significance. For example: "The predicted binding of cidofovir to ASNS suggests that existing antiviral drugs may have off-target effects on host metabolic enzymes."**

Answer: Thank you for this suggestion. The revised discussion focuses on conceptual integration, removing redundant technical details and emphasizing biological implications (e.g., cidofovir’s off-target effects).

Location in Revised Manuscript: Discussion section, Lines 534-551; Lines 587-589

**6.II Question: Need to adhere to the principle of using "correlation" rather than "causal relationship" expressions. For example, the idea of a "self-sustaining loop" between ASNS and PI3K-AKT-mTOR is attractive but only based on correlation analysis, which may lead to overinterpretation. It is recommended to use cautious language such as "may suggest" or "hypothetical."**

Answer: We appreciate your reminder. We used cautious language (e.g., "may point to a potential reciprocal regulatory loop") to describe correlative findings, avoiding causal overstatements.

Location in Revised Manuscript: Discussion section, Lines 558-562

**6.III Question: Although immune checkpoints such as LAG3 are mentioned, they are not deeply linked to the main research line of ASNS/metabolic signaling pathways. It is recommended to explicitly connect immune checkpoint regulation to ASNS/PI3K-mTOR activity and viral immune evasion strategies.**

Answer: Thank you for highlighting this gap. We linked LAG3 to ASNS-PI3K-AKT-mTOR activation, proposing it as a downstream immune evasion mechanism coordinated with metabolic hijacking.

Location in Revised Manuscript: Discussion section, Lines 568-575

**6.IV Question: The association between ASNS expression in plasma cells and increased CMV susceptibility in HIV-positive patients is valuable but highly speculative. The expression should be presented rigorously.**

Answer: We are grateful for your feedback. We present this association rigorously, noting no explicit CMV coinfection data in the cohort and framing it as a speculative link requiring validation.

Location in Revised Manuscript: Discussion section, Lines 576-584

**6.V Question: The cidofovir binding data are significant, but overemphasizing their clinical application value is inappropriate without in vitro or in vivo validation. In general, cautious language such as "suggests," "consistent with," or "may represent" should be used to avoid absolute claims.**

Answer: Thank you for this reminder. We used cautious language to describe cidofovir’s potential, framing it as a "preliminary framework" and noting the need for in vitro/in vivo validation.

Location in Revised Manuscript: Discussion section, Lines 589-591

**[6.VI](6.VI) Question: Need to clearly define the innovation of this study, emphasizing that this is the first time ASNS has been identified as a metabolic-signaling hub in CMV/HIV coinfection through integrated analysis.**

Answer: We appreciate your suggestion. The discussion’s opening explicitly highlights our novelty: this is the first study to identify ASNS as a core metabolic-signaling hub in CMV/HIV coinfection via integrated multi-dataset analyses.

Location in Revised Manuscript: Discussion section, Line 534

**6.VII Question: It is recommended to divide the discussion into 4-5 thematic subsections, including: (1) ASNS as a metabolic-signaling hub in viral infection; (2) Interaction with the PI3K-AKT-mTOR pathway and its significance for viral pathogenesis; (3) Immune evasion and plasma cell-specific expression; (4) Therapeutic potential and limitations; (5) Future directions (e.g., multi-omics research and functional validation).**

Answer: Thank you for this framework. We restructured the discussion into thematic subsections aligned with your recommendation, enhancing conceptual flow.

Location in Revised Manuscript: Discussion section, Lines 533-623 (thematically divided paragraphs)

**7. Conclusions**

**7.I Question: This section resembles a simple review of results, repeating technical details (e.g., VAL-51, ASN-74 sites, number of transcription factors) without extracting core conceptual insights. It is recommended to move details such as binding sites and transcription factor names to the results/discussion sections, and focus on conceptual advances and significance in the conclusions.**

Answer: Thank you for this guidance. The revised conclusion focuses on conceptual advances (ASNS’s hub role, translational potential) and removes redundant technical details.

Location in Revised Manuscript: Conclusions section, Lines 625-641

**7.II Question: Given that the study results are only based on correlation analysis and in silico simulations, expressions such as "elucidated the key role" are overly absolute. It is recommended to use cautious language such as "suggests a core role" or "identifies ASNS as a candidate hub."**

Answer: We appreciate your feedback. The conclusion uses cautious language aligned with our correlative/in silico data, avoiding absolute expressions.

Location in Revised Manuscript: Conclusions section, Line 637

**7.III Question: The conclusion does not return to the broader research context (e.g., viral exploitation of host metabolism, new paradigms in coinfection biology, significance for precision virology). It is recommended to link the study findings to broader themes such as viral metabolic hijacking, systems biology of coinfection, and opportunities for host-directed therapy.**

Answer: Thank you for this suggestion. The revised conclusion links findings to broader themes (viral metabolic hijacking, precision virology) to contextualize significance.

Location in Revised Manuscript: Conclusions section, Lines 625-628

**7.IV Question: Expressions such as "targeted therapy and personalized medicine strategies" are overstated and should be adjusted to hypotheses requiring future validation.**

Answer: We are grateful for your feedback. We revised therapeutic descriptions to frame ASNS as a "promising experimental target," emphasizing the need for future validation.

Location in Revised Manuscript: Conclusions section, Lines 635-638

**7.V Question: Emphasize the innovation of the study, explicitly stating that this is the first time ASNS has been nominated as a metabolic-signaling hub in CMV/HIV coinfection through integrated analysis.**

Answer: Thank you for this reminder. The conclusion explicitly states we are the first to identify ASNS as a metabolic-signaling hub in CMV/HIV coinfection via integrated bioinformatics.

Location in Revised Manuscript: Conclusions section, Line 625

**[7.VI](7.VI) Question: Conclude with a forward-looking statement, such as calling for experimental validation, multi-omics research, and translational medicine exploration.**

Answer: We appreciate your suggestion. The conclusion specifies future priorities: ASNS pathway validation, multi-omics integration, and clinical evaluation of the ASNS-RUNX1 axis.

Location in Revised Manuscript: Conclusions section, Lines 639-641

We want to express our deepest gratitude to all Reviewers for your meticulous efforts, profound expertise, and invaluable feedback! Your careful scrutiny of every detail—from conceptual framework to technical nuances—and your thoughtful suggestions have been instrumental in elevating the rigor, clarity, and impact of our manuscript. The time and care you invested reflect a true commitment to scientific excellence, and we are genuinely grateful for your guidance. We hope the revised work honors the dedication you put into helping us improve.

Sincerely,

Wei Lu, PhD

Corresponding Author

Geriatrics Center, Wuxi Second Geriatric Hospital

Wuxi, Jiangsu Province, China

Email: [luwei5166@gmail.com](mailto:luwei5166@gmail.com)
